# Nursing Students' Views on the Timing of Simulation-Based Education: Before Versus During Clinical Placements

**DOI:** 10.7759/cureus.88342

**Published:** 2025-07-20

**Authors:** Walaa H Maghrabi, Shahad A Alshehri, Wajan N Alqahtani, Remas Z Alshehri

**Affiliations:** 1 Department of Maternity and Child Health Nursing, King Abdulaziz University, Jeddah, SAU

**Keywords:** education, healthcare simulation, nursing, simulation in medical education, students

## Abstract

Background: Simulation-based education is widely recognized for enhancing nursing students’ clinical preparedness and confidence. However, limited research has explored how the timing of simulation, before vs. during clinical placements, affects perceived effectiveness. This study aimed to explore nursing students’ perceptions of the effectiveness of simulation-based training delivered at two different time points: before and during clinical placements.

Methods: A within-subject cross-sectional survey was conducted among 125 final-year undergraduate nursing students at a university in Saudi Arabia. The survey included quantitative items and an open-ended question to capture perceptions. Descriptive statistics, paired samples t-test, and qualitative content analysis were used. The study followed Strengthening the Reporting of Observational Studies in Epidemiology cross-sectional reporting guidelines.

Results: Although 76 students (60.8%) preferred preclinical simulation, positioning it as a critical preparatory step in clinical readiness, there was no statistically significant difference in effectiveness ratings between the two timings (mean = 6.5, p = 0.80; Cohen’s d = 0). Qualitative responses showed that students valued both timings for distinct purposes: preclinical simulation reduced anxiety and built foundational skills, while in-placement simulation reinforced learning and supported real-time application. A combined simulation approach was favored by 101 students (80.8%), supporting the value of longitudinal integration.

Conclusion: Students perceive simulations as beneficial, regardless of timing, but recognize the unique value of each. Findings highlight the importance of strategically and flexibly embedding simulation across the curriculum. Nursing programs should adopt a developmentally sequenced simulation strategy that supports learners’ progression and addresses both preparatory and reinforcing roles across the clinical education continuum.

## Introduction

Simulation-based education has become a core component of nursing curricula, offering students a safe, structured, and interactive environment to develop essential clinical skills before encountering real patients. It enhances critical thinking, promotes clinical decision-making, and bridges the gap between theoretical knowledge and practical application [1-3]. Additionally, simulation provides opportunities for repetitive practice, immediate feedback, and structured debriefing, all of which are essential for mastering complex procedures and clinical judgment [4,5]. These features help learners build confidence, reduce anxiety, and improve readiness for real-world patient care [6].

Beyond its foundational role in skill development, simulation offers distinct advantages that further justify its integration into healthcare education. One key benefit is its ability to replicate rare or high-risk clinical scenarios that students may not routinely encounter, ensuring comprehensive clinical preparation [5,7]. Simulation also supports interprofessional learning by fostering collaboration across healthcare disciplines and promoting patient-centered care [8]. A growing body of research confirms that simulation-based training enhances clinical competence, improves patient safety, and leads to better clinical outcomes than traditional methods [9,10]. Notably, a landmark study by the National Council of State Boards of Nursing found that up to 50% of traditional clinical hours can be effectively replaced with simulation without compromising licensure or clinical performance outcomes [11]. The COVID-19 pandemic further accelerated the integration of simulation into nursing programs, as clinical site access became limited and institutions relied more heavily on simulation to meet educational requirements [12,13].

Despite the well-established benefits of simulation in healthcare education, limited evidence exists on the optimal timing of its delivery within the clinical training continuum. Most studies assess simulation as a general teaching strategy, often overlooking whether outcomes differ when implemented before vs. during clinical placements. This study addresses that gap by examining nursing students’ perceptions of simulation effectiveness at these two time points. Specifically, it explores the following: Does simulation conducted prior to clinical practice enhance students’ preparedness and confidence more than simulation delivered during clinical placements? Do students perceive combining both timings as more effective than using either alone?

## Materials and methods

Study design

This study used a quantitative, within-subject cross-sectional design to compare nursing students’ perceptions of simulation conducted before and during clinical placements. The within-subject approach allowed each participant to serve as their own control, thereby minimizing the effect of interindividual variability on the comparison between the two simulation timings. The study adhered to Strengthening the Reporting of Observational Studies in Epidemiology cross-sectional reporting guidelines [14].

Sample and setting

This study was conducted at King Abdulaziz University, a public university in Saudi Arabia, targeting final-year undergraduate nursing students. These students were selected because they had experienced simulation-based training at two distinct points in their curriculum: 1) before clinical placements, and 2) during clinical placements. Using simple random sampling, a required sample size of 123 students was calculated from a population of 180 using Cochran’s formula (95% confidence level, 5% margin of error). Inclusion criteria included current enrollment in the final year of the nursing program and willingness to complete the online questionnaire.

Data collection

Data were collected between February 24, 2025, and March 10, 2025, using a self-administered online questionnaire developed through Google Forms (Google LLC, Mountain View, CA). The survey link was distributed via students’ official institutional email accounts and shared through the academic WhatsApp group (WhatsApp Inc., Mountain View, CA) designated for final-year nursing students to maximize participation. The invitation message explained the study’s purpose, included a confidentiality statement, and presented a consent statement that required participants to indicate their agreement before accessing the questionnaire. Only those who selected “Agree” could proceed. Participation was entirely voluntary and anonymous; no identifying information was collected.

Study measures

Data were collected using a researcher-developed online questionnaire created specifically for this study to explore nursing students’ perceptions of simulation-based training delivered at two time points: before and during clinical placements (see the Appendix). The questionnaire design was informed by prior literature on simulation-based learning in nursing education [2,15].

To establish content validity, two faculty experts in nursing education and simulation reviewed the questionnaire for clarity, relevance, and alignment with the study aims. Additionally, cognitive interviews were conducted with four nursing students from the target population to assess item interpretation and identify any ambiguities. Minor wording revisions were made based on feedback from both experts and students to enhance clarity and consistency.

Because the questionnaire was developed for a specific, descriptive purpose and did not include multi-item scales or attempts to measure latent constructs, further psychometric testing (e.g., construct validity and internal consistency) was not applicable. However, the within-subject design contributed to internal validity by allowing direct comparison across the two simulation timings within the same participants, minimizing individual variability.

The questionnaire consisted of two main sections. The first section gathered demographic information (e.g., gender and monthly household income). The second section included simulation-related items that addressed students’ perceptions of simulation timing. These included questions such as the following: “In general, do you find simulation training helpful in improving your clinical preparedness and confidence?” with response options of Yes, No, or Neutral; and “Which timing do you find more effective in improving clinical preparedness and confidence?” with options including Before clinical practice, During clinical practice, Neutral, or Neither effective. Students were also asked an open-ended question: “In your opinion, what is the ideal timing for conducting simulation training and why?”

To address Research Question 1, participants rated their perceived preparedness and confidence using a 0-10 scale (where 0 = no readiness/confidence and 10 = maximum readiness/confidence) in response to two items: one evaluating the simulation conducted before clinical placement and the other evaluating the simulation conducted during clinical placement. To address Research Question 2, students were asked whether they believed combining both timings, before and during clinical practice, was more effective than using either timing alone, with response options of Yes, No, or Neutral.

Data analysis

Data were analyzed using STATA version 17 (StataCorp LLC, College Station, TX). Descriptive statistics were used to summarize all study variables. A paired sample t-test was conducted to compare students’ perceived effectiveness of simulation before and during clinical placements, with effect size (Cohen’s d) calculated to assess the magnitude of the difference. Statistical significance was set at p < 0.05.

The questionnaire included one open-ended item asking students to indicate their preferred simulation timing and explain their rationale. Responses were analyzed using basic content analysis [16] to identify common patterns in students’ reasoning. The first author conducted an initial review and grouped responses by timing preference and purpose. The remaining three authors independently reviewed the categorization to ensure clarity, consistency, and consensus. This focused qualitative analysis was intended to complement the quantitative findings and provide additional insight into students’ perceptions.

## Results

A total of 125 final-year undergraduate nursing students completed the survey. To ensure data completeness, responses to all items were required, resulting in no missing data. The participants had a mean age of 21.6 years (SD = 0.9). The average grade point average (GPA) was 4.3 out of 5 (SD = 0.4). Most of them were female participants (72%), and 28% were male participants. Monthly household income varied, with the largest group (28.8%) reporting 10,000-15,000 Saudi Arabian Riyal (SAR), followed by 24% reporting 21,000 SAR or more. The majority (79.2%) found simulation training useful for improving clinical preparedness and confidence, while 8% did not. Most students (60.8%) preferred simulation before clinical placements, and 19.2% favored simulation during placements (Table 1).

**Table 1:** Characteristics of the sample SAR: Saudi Arabian Riyal

Variables (n = 125)	Frequency	Percentage
Gender		
Male	35	28%
Female	90	72%
Monthly income (SAR)		
Less than 5,000	13	10.4%
5,000-9,000	17	13.6%
10,000-15,000	36	28.8%
16,000-20,000	29	23.2%
21,000 or more	30	24%
Usefulness of simulation in general		
Useful	99	79.20%
Not useful	10	8%
Neutral	16	12.8%
Effective timing of simulation		
Before clinical practice	76	60.8%
During clinical practice	24	19.2%
Neutral	19	15.2%
Neither effective	6	4.8%

Open-ended responses revealed that students valued both timings for distinct yet complementary educational purposes: preclinical simulation functioning as an entry point for building foundational skills and reducing anxiety, and in-placement simulation was valued for reinforcing learning and addressing real-time challenges. Importantly, students endorsed a combined approach, highlighting the importance of a developmentally sequenced simulation curriculum that aligns with learners’ evolving competencies, needs, and stages of clinical exposure (Table 2).

**Table 2:** Content analysis of the open-ended question

Purpose	Preferred timing	Representative quotes	Interpretation
Clinical preparedness and anxiety reduction	Before clinical placement	“Simulation before clinical helps us practice in a safe space and gain basic skills before facing real patients.” “It reduced my anxiety and made me feel more confident entering the hospital.”	Preclinical simulation was most frequently associated with clinical preparedness and anxiety reduction, establishing it as a foundational component.
Skill reinforcement and reflective practice	During clinical placement	“Having simulation during clinical helped me apply what I saw and correct my mistakes.” “Simulation while on placement helped me reflect and improve continuously.”	Intraclinical simulation was valued for skill reinforcement and addressing real-time challenges
Continuous learning and integration	Combined timing	“Both are important, before placements prepare me, and during placements help me improve.” “Some courses need simulation early, others during the clinical part, it depends.” “Instead of simulation being a one-time phase, students should have individualized simulation plans, extra sessions if needed, or faster transition to clinicals if ready.”	The endorsement of a combined approach reflects students' recognition that each simulation timing offers distinct yet complementary educational purposes and should be developmentally sequenced and integrated across the nursing curriculum

Comparison of perceived effectiveness of simulation timing (before vs. during clinical placements)

Participants rated their preparedness and confidence on a 0-10 scale for both simulation timings. Mean scores were identical: 6.5 before placements (SD = 2.7) and 6.5 during placements (SD = 2.5). A paired sample t-test showed no significant difference, t(124) = -0.1, p = 0.80, with a negligible mean difference (-0.4; 95% CI: -0.5 to 0.5) and an effect size (Cohen’s d) of 0. These results indicate that students perceived both timings as equally effective (Table 3). While the mean scores were identical, the mid-range averages and relatively large SDs suggest that ceiling or floor effects are unlikely. The absence of a significant difference likely reflects genuinely similar perceptions of the two simulation timings rather than limitations of the rating scale.

**Table 3:** Perceived effectiveness of simulation timing (before vs. during clinical placements) This table addresses the research question: Does simulation conducted before clinical placements enhance students’ preparedness and confidence more than simulation during placements? Participants rated their preparedness and confidence for each timing on a 0-10 scale (0 = no readiness/confidence; 10 = maximum readiness/confidence). A paired sample t-test was used to compare the perceived effectiveness of the two conditions SD: standard deviation; df: degrees of freedom

Variable (n = 125)	Mean	SD	95% CI	t	df	p
Simulation before clinical placement (0-10)	6.5	2.7	6 to 7	-	-	-
Simulation during clinical placement (0-10)	6.5	2.5	6.1 to 7.3	-	-	-
Difference	-0.4	3.0	-0.5 to 0.5	-0.1	124	0.8

Perceived value of combined simulation timing

The majority of students (80.8%) believed combining simulation before and during clinical practice is more effective than using either alone, indicating strong support for an integrated approach across the clinical education continuum (Table 4).

**Table 4:** Perceived value of combined simulation timing This table addresses the research question: Do nursing students perceive combining simulation before and during clinical practice as more effective than using either timing alone? Participants responded to a single-item question asking whether a combined simulation approach is more effective. Responses were reported as frequencies and percentages

Variable (n = 125)	Frequency	Percentage
Combined simulation timing		
Yes (more effective than using either timing alone)	101	80.8%
No (less effective than using either timing alone)	11	8.8%
Neutral	13	10.40%

## Discussion

This study investigated nursing students’ perceptions of the effectiveness of simulation-based training conducted before and during clinical placements. While a greater proportion of students (60.8%) expressed a preference for pre-clinical simulation, positioning it as a critical preparatory step in clinical readiness, there was no statistically significant difference in effectiveness ratings between the two timings. Both conditions received identical mean scores (6.5), and the calculated effect size (Cohen’s d = 0) indicated no practical difference. These results suggest that nursing students view simulation as an essential component of clinical preparation, regardless of whether it is implemented before or during clinical placements.

Importantly, responses to the open-ended question provided a more nuanced perspective on simulation timing. Simulation conducted before clinical placements was associated with reducing anxiety, building foundational skills, and increasing confidence as students transitioned into the clinical environment. These perceptions are consistent with previous research, which has shown that early simulation exposure supports psychological safety and readiness for practice [17-19]. In contrast, simulation during clinical placements was valued for reinforcing learned content, addressing gaps in performance, and applying skills in real-time, findings that align with literature positioning simulation as a reflective tool embedded within clinical learning [10,15].

These findings are supported by the NLN Jeffries simulation theory, which emphasizes contextual alignment, learner-centered design, and developmental sequencing in simulation-based education [3]. According to the theory, simulation should be strategically embedded across the curriculum to address learners’ evolving needs, from introductory skills to advanced clinical decision-making. In this context, simulation prior to clinical placements can serve as a preparatory platform for competence building, while in-placement simulation enhances the application, synthesis, and refinement of clinical skills in practice.

The strong student endorsement of a combined simulation approach, reported by over 80% of participants, further underscores the value of longitudinal integration of simulation across multiple stages of the curriculum. A growing body of research supports the notion that repeated, scaffolded simulation experiences, delivered with increasing fidelity and complexity, are more effective than isolated sessions [9,20]. This educational model aligns with constructivist learning theory, which posits that learners construct knowledge progressively through active engagement with developmentally appropriate tasks [21]. Therefore, simulation should not be viewed as inherently more effective at a specific point in time, but rather as a longitudinal, integrated pedagogical strategy that evolves with the learner. In this context, both preclinical and intraclinical simulation have distinct and complementary roles, and their combined use, strategically sequenced and embedded across clinical phases, offers the greatest potential for enhancing clinical preparedness and competence.

Implications for healthcare education

The results of this study underscore the educational value of simulation when incorporated at multiple points throughout clinical training. Rather than being confined to a single stage, simulation appears most effective when applied as part of a continuous learning process that aligns with students’ developmental progression.

Embedding simulation throughout the curriculum, with increasing complexity and clinical relevance, can better support skill acquisition and readiness. The lack of significant difference in perceived effectiveness between pre- and intraclinical simulations indicates flexibility in scheduling, allowing adaptation to institutional resources. Nevertheless, adjusting simulation content to match the learner’s stage, emphasizing confidence-building early on and performance refinement later, may optimize its benefits.

The strong student preference for a combined approach highlights the value of a flexible, student-centered simulation strategy that accommodates diverse learning needs. A tailored and sequenced simulation curriculum that evolves with learners’ progression may better support student competence and readiness for professional practice.

Limitations and strengths

This study has some limitations that should be considered when interpreting the findings. First, the study relied on students’ self-reported perceptions of simulation effectiveness. While this provides valuable insight into the learner’s experience, such subjective data may not fully reflect actual clinical competence. Future research should include objective measures of clinical performance. Second, the sample was drawn from a single academic institution, which may limit the generalizability of the findings to other educational settings with different curricula, faculty expertise, or simulation infrastructure. Third, this study employed a self-developed questionnaire, as no standardized tool was available for assessing students’ perceptions of simulation timing. Although the tool was not adapted from a validated scale, its development was guided by relevant literature and informed by expert review and cognitive interviewing. This approach ensured contextual relevance and alignment with the study’s objectives. Nonetheless, the use of a study-specific instrument may limit comparability with studies employing broader or differently framed simulation evaluation tools.

A notable strength of this study is its within-subject design, which allowed each participant to serve as their own control when comparing simulation before and during clinical placements. This approach helped to control for individual variability in key characteristics such as gender, GPA, baseline confidence, learning preferences, and clinical exposure. As a result, potential confounding variables were minimized, enhancing the internal validity of the study’s comparisons.

## Conclusions

The findings highlight simulation-based training as an indispensable component of healthcare education. Regardless of the timing, students consistently viewed simulation as an effective means of enhancing their clinical preparedness and confidence. Integrating simulation throughout the clinical continuum, particularly both before and during clinical placements, can best enhance students’ confidence, competence, and continuity of learning. The strong preference for a combined approach further supports simulation as a longitudinal strategy rather than a standalone intervention. These findings support a broader curricular shift toward simulation-rich curricula that foster clinical reasoning, safe practice, and professional readiness.

## Appendices

Study questionnaire

Section 1: Demographic Information

How old are you? ________ years

What is your gender? ☐ Male ☐ Female

What is your current cumulative grade point average? ________ (e.g., 4.3)

What is your household’s average monthly income in Saudi Riyals? ☐ Less than 5,000 ☐ 5,000-9,000 ☐ 10,000-15,000 ☐ 16,000-20,000 ☐ 21,000 or more

Section 2: Simulation-Related Items

In general, do you find simulation training helpful in improving your clinical preparedness and confidence? ☐ Yes ☐ No ☐ Neutral

Which timing do you find more effective in improving clinical preparedness and confidence? ☐ Before clinical practice ☐ During clinical practice ☐ Neutral ☐ Neither effective

In your opinion, what is the ideal timing for conducting simulation training and why? Please write your response below.

Simulation Timing (Before vs. During Clinical Placements)

Please rate your confidence and preparedness for clinical practice following each type of simulation on a scale from 0 to 10, where 0 = not at all confident/prepared and 10 = extremely confident/prepared.)

How prepared and confident did you feel for clinical practice after participating in a simulation conducted before your clinical placement?

Scale: 0 ☐ 1 ☐ 2 ☐ 3 ☐ 4 ☐ 5 ☐ 6 ☐ 7 ☐ 8 ☐ 9 ☐ 10

How prepared and confident did you feel for clinical practice after participating in a simulation conducted during your clinical placement? Scale: 0 ☐ 1 ☐ 2 ☐ 3 ☐ 4 ☐ 5 ☐ 6 ☐ 7 ☐ 8 ☐ 9 ☐ 10

Combined Simulation Timing

Do you believe combining both timings (simulation before and during clinical practice) is more effective than using either timing alone? ☐ Yes ☐ No ☐ Neutral
